# The Remedial Value of Mercury

**Published:** 1872-12

**Authors:** Lunsford P. Yandell

**Affiliations:** Louisville, Kentucky


ATLANTA

### MEDICAL AND SURGICAL JOURNAL.

VOL. X.—DECEMBER, 1872.—NO. 9.

---

### ORIGINAL COMMUNICATIONS.

---

### THE REMEDIAL VALUE OF MERCURY.

BY LUNSFORD P. YANDELL, M.D.,
*Louisville, Kentucky.*

The alchemists were acquainted with seven metals, which they named after the sun and moon, and the five planets then known to astronomers. The terms saccharum, saturni, salmatis, and lunar caustic, which are still occasionally used, denote their origin, and in the old pharmacopœias one of the preparations of copper is described under the fanciful name of *ens veneris;* but mercury is the only metal which retains its alchemical name. It has been known from a high antiquity, but no use of it had been made in medicine before the times of Hippocrates, in whose writings it is not mentioned. Aristotle and Dioscorides refer to it as poisonous in its nature, and Galen speaks of it as possessing corrosive properties. Messue, an Arabian physician, was the first to venture upon its use in diseases, and his practice was limited to the application of the remedy to affections of the skin. Avianna discovered the interesting fact that mercury might be swallowed with impunity, and that the metal passed unchanged through the intestines. It was about the end of the thirteenth century that mercurial preparations were introduced into Europe. They were regarded for a long time as dangerous remedies, and gained little favor with the profession until the venereal disease was found to yield to them, and their action in that disease attested the safety with which they might be administered. The first of all mercurials that obtained a substantial footing with scientific physicians, was a pill composed of mercury, rhubarb and musk—the *pilula barbarossæ.*

Mercury was tortured by the alchemists in their search after the philosopher's stone, by every process known to the science of their times. It was subjected to intense heat, and by its reaction on sulphur and common salt, in the hope of fixing the metal, they discovered some of its most active compounds. The chemistry of a later day has added to the number of these preparations, while the science of therapeutics has been carefully investigating their remedial action. They have been applied in the treatment of disease in almost every conceivable circumstance and in every form—as ointments, vapors, caustics, emetics, cathartics, alterants, anthelmintics. They are still held in high repute as the most efficacious remedy for syphilis, and it is only a little while ago that they claimed the first rank among our febrifuges. The class "sialagogues," is no longer retained in our systems of materiæ medica, but so long as it was deemed good practice to salivate, no medicine could compare in efficacy with mercury.

As a remedy, mercury has experienced every variety of fortune. It has been at the same time extolled as a panacea and denounced as poison by opposite sects in the profession. Those who first employed it were styled "quacks," from its name, quacksalber, and the term has come down to us as one of reproach. It has been by turns the most popular and the most odious of medicines. In the course of my professional life I have seen the ups and downs of many remedies, but in all my medical experience I have witnessed nothing so strange as the history of mercury. Fifty years ago it would have been very difficult to find in this country a practitioner who did not place it in the foremost rank of our therapeutic agents. To-day, it would perhaps be impossible to find one, in any country, who would claim for it such a place, while not a few insist that it should be discarded altogether. I speak particularly of calomel, the most extensively used, as it has been also the most abused, of all the mercurial compounds. Nor need we hesitate to admit that in the whole history of medicine, we read of no greater abuses than those which have attended calomel. The medicine has been given in every character, grade and form of almost every disease, and no doubt often in the hope, which has sometimes been exposed, that the subtle agent would reach the complaint of which the physician could not make out the diagnosis. And so it has been given when the nature of the malady was known and when it was not known, and when nothing else has availed it has been common to turn to it as the last resort.

My first instructions in the administration of calomel, derived from my father, who had adopted the practice of Sydenham and Cullen, were eminently cautious; so much so, indeed, that they appeared to me timid, if not trifling, when I came, a few years afterwards, to listen to my medical teachers in Lexington and Baltimore. Accepting the views of Johnson and other writers on the diseases of the tropics, the professors in those schools taught us to give calomel by the scruple and the drachm. Dr. Cooke, at a later day, improved upon this heroic practice and gave it to his patients, during the prevalence of cholera in Lexington, in 1833, in doses of two ounces. To a young man, aged nineteen, he gave a table spoonful every six hours for three days; and to another young man, who recovered from the disease, he gave ten table spoonfuls in two days. This patient, Dr. Cooke remarks, " was somewhat salivated." In the case which resulted in death, the calomel passed in balls, enveloped in mucus, through the bowels, as in fact it did in all cases where it was administered in such quantities, for being almost insoluble far the larger portion necessarily escaped unaltered.

This practice was attended by a serious drawback, the salivation of most of the patients who recovered, in many instances the ptyalism being distressing; and perhaps nothing tended so much to bring mercury into disrepute as these accidents. But for a long time salivation was regarded by physicians as eminently curative. It was remarked that patients in acute diseases were pretty sure to show signs of convalescence so soon as ptyalism appeared, and the inference was natural that the ptyalism cured them. The practitioner was, therefore, never so well pleased as when he found that the gums of his patients were touched, and he sought in every way to bring about this state of the system, rubbing mercurial ointment into the thighs, as well as giving calomel freely by the mouth. Of few things in my experience as a medical student is my recollection more vivid than of patients treated upon this plan by my old preceptor, Professor Potter, in Baltimore. I recall with great distinctness the fetid wards in the infirmary near the university—rendered fetid by the mercurial breath of the patients—the rows of beds stretching down them, and their cachectic occupants, with swollen faces, and bowls under their chins, and the saliva-flowing in streams from their mouths. I had not seen a similar practice in Lexington, the winter before, simply because there was then no hospital in which to carry it out. But this was the practice of our country fifty years ago.

The fear of salivation from mercury has always been present to the minds of the people, and such abuse of the powerful remedy could not end otherwise than in a reaction against it. The circumstances which hastened the revolution which has occurred in professional opinion concerning it, need not here be stated. It is enough that it has been complete—that from one extreme we have run to the other—that we have teachers who once gave it with the boldest hand, now declaring that it is a medicine to be prescribed with fear and trembling. But the question obtrudes itself upon us: is there wisdom in this extreme movement? Is the revolution based upon experience and a sounder pathology? Were the therapeutics of our fathers in medicine so pernicious?

I should be sorry to answer this last question in the affirmative. I should be pained to believe that medicine had been cultivated so long, and with such care, in vain. But I would fain hope that we are gaining upon the darkness, and that we understand better than our predecessors the limits under which mercurials should be prescribed. Happily we are in possession of febrifuges which leave us without excuse for the administration of calomel, in a large class of diseases. A sounder pathology has shown that to many inflammatory affections, in which it was formerly employed, it is not adapted; and experience has fully refuted the notion that salivation corrects morbid actions.

The danger from calomel is salivation. If this be escaped, the mischief from it is never serious. It never induces excessive purgation, even in the largest doses, and as to that secret poisoning of the system which has been charged to it, showing itself in the bones after the lapse of years, my belief is that the suspicion is wholly unfounded. But salivation is an accident greatly to be deprecated, particularly in young persons, to whom it may prove fatal, or the cause of deformities hardly less frightful than death. For this reason calomel should be given with the greatest caution to children who have completed their first dentition. It is preëminently the medicine for infants; but we cannot too narrowly watch its action in children after the period alluded to. Among this class of patients have occurred the most melancholy examples of its destructive power. In women, too, it is well understood that ptyalism is readily excited, and it is always a good rule before prescribing calomel to a female patient to ask her whether she has ever been salivated. No practitioner who values his reputation will give it to women or children without returning in due time to see that it has acted upon the bowels. This is the one essential precaution to be observed in the use of calomel. Even with its observance, we shall still now and then salivate some of our female patients; but if the dose be small and its action free, the resulting ptyalism will not be formidable, though it is always annoying. The evil is very much greater in children, and the danger of it was a fruitful source of anxiety to the physician when it was customary to give calomel in almost all their complaints.

The class of disorders in which mercury has been held to exert the happiest effect are complaints of the liver—affections in which biliary secretion was supposed to be suspended, impaired, or disordered. For a century, at least, this was the accepted doctrine of the medical world. If not a purger of bile, mercury was nothing. It was no more doubted that calomel acted upon the liver than that opium allayed pain. But this ancient opinion has lately been called in question, and experiments on dogs have been cited to show that the mercurial, instead of promoting biliary secretion, really diminishes the discharge of bile. Without stopping to criticise these obervations, I will venture the opinion that the clinical experience of a hundred years is worth more to the practitioner than a vast number of experiments upon the lower animals; and that whether the matters voided after a dose of calomel be bilious or the excretions of other viscera than the liver, the relief which follows its action in many cases is such as results from the operation of no other purgative medicine. So that whether we hold to the old idea of a cholagogue action from it, or agree that it relieves by the elimination of other matter, the fact remains that its therapeutic effects in disorders usually regarded as bilious are unique. There is a disordered state of the system which seems to be connected with torpor of the liver, indicated by acidity of stomach, indigestion, headache, and general dullness of mind and body, which is corrected more certainly by calomel than by any other remedy. Dr. James Johnson, so long editor of the *Medico-Chirurgical Review*, was in the habit of saying that his mind was never so clear, and he was never so capable of intellectual effort as after pumping out his liver by twenty grains of calomel. My own experience is similar with regard to the calomel, rhubarb, and aloes pills, identified with the fame of my old friend, Dr. Cooke.

In all the varieties of cholera, the Asiatic, the sporadic, and that of children, this medicine appears to me indispensable in all but the mildest cases; and when an epidemic cholera is raging I should certainly not permit a case of diarrhœa to run on a single hour without resorting to calomel. Nor should I consider a case of the disease cured until calomel had restored what we have been accustomed to regard as biliary secretion. I may add, with perfect assurance, that I never saw a case of this malignant disease terminate fatally after these evacuations had been established. The sporadic cholera, of which we see cases every summer, yields more certainly to it than to any other treatment I have ever pursued; and my experience is equally favorable to it in cholera infantum.

The phlegmasiæ, in which the action of mercury was for a long time looked upon as peculiarly benign, are now seldom treated by this medicine, and it cannot be doubted that the change of therapeutics is for the better. Few physicians at this time give calomel in rheumatism, pneumonia, pleurisy, or peritonitis, or if it is given, it is simply as a mild purgative and in no hope of shortening the disease. In dysentery it is oftener prescribed, and many physicians insist with advantage, but the experience of years has not impressed me favorably with it in this complaint.

As a vermifuge, mercury, from an early day, has enjoyed a high reputation. Looking through the works of the learned Boyle, a short time since, I met with the history of a case illustrative of its action in this, which is curious, and I am sure will be read with interest. It is as follows:

"A fine boy, born to be heir of a very illustrious family, falling into a dangerous fever, judged to be from worms, or verminous matter, the famous and experienced physician who treated him confessed that he had no hope of him, because the child, bred to have his will, and being tired of unsuccessful remedies, was so obstinate as to drink nothing but small beer. Whereupon I advised that his beer be impregnated with mercury by shaking quicksilver therein. By which means the patient, perceiving no change of color or taste in his drink, swallowed it greedily and soon after recovered."

In the malarial fevers, such is the efficacy of quinine that calomel is now seldom thought of as a remedial resource; and yet in remittent fever I strongly incline to the opinion that its action tends to bring about an intermission, and I am confident that it greatly promotes the comfort of the patient. Bnt if its use must be conjoined with the prohibition of cold water, as was once the custom, I certainly should never recommend it in any febrile affection, cold drinks and cool ablutions possessing a value in such complaints which no one would now claim for calomel.

I suppose the medical world is nearly agreed that mercury is our best anti-syphilitic, though there are those who profess to have found another mode of treating the venereal disease with more safety and equal success. The most efficacious method seems to be the vapor bath, which possesses the advantage of not salivating the patients to any serious extent.

In regard to the proper dose of calomel the profession is not now much divided. We all incline to small doses. And it seems wonderful that practitioners could ever have expected effects from this medicine proportioned to the amount given, considering its extreme insolubility. A few grains will produce the purgative action of as many scruples, only a very small quantity undergoing solution in the intestines. I have taken it in doses of sixty grains with the effect of inducing a single passage, and again have had six evacuations from ten grains, the difference in its action depending upon the state of the system at the time. There is therefore no advantage in the excessive doses which it was once the practice to give, and there is this objection to them, that enough of the mercurial may be detained in the alimentary canal to excite a serious ptyalism. I constantly prescribe it to young infants, in doses of a quarter or half a grain, and children with the summer complaint will take it, day after day, in two or three grain doses with benefit; but if I deem it proper to prescribe it to children of a greater age at all, I am careful not to repeat it often, and give only in grain, or two grain doses.

I will not say that calomel is now too little employed in the practice of medicine in our country; perhaps the majority of physicians still resort to it often enough; but there can be no doubt that there is a growing prejudice against it in the profession. I am free to confess that I do not myself rank it so high among remedial agents as I did when I began the practice of physic. I am far from prescribing it so often, and I give it in smaller quantities. My conviction in the earlier years of my professional life was that I could not practice a day without calomel. I certainly am unable still to see how I could get on without it in many diseases. I still esteem it one of our best remedies, and in many morbid conditions to which I have not had time to allude in this short paper; but I find the class of diseases a growing one in which it no longer appears to me an indispensable remedy.

December 1st, 1872.

